# Global longitudinal strain is superior to ejection fraction for long‐term follow‐up after allogeneic hematopoietic stem cell transplantation

**DOI:** 10.1002/jha2.586

**Published:** 2022-11-07

**Authors:** Marika Watanabe, Kimikazu Yakushijin, Hidekazu Tanaka, Ruri Chijiki, Miki Saeki, Yuri Hirakawa, Hidetomo Takakura, Yutaro Usui, Hiroya Ichikawa, Rina Sakai, Sakuya Matsumoto, Shigeki Nagao, Yu Mizutani, Keiji Kurata, Akihito Kitao, Yoshiharu Miyata, Yasuyuki Saito, Shinichiro Kawamoto, Katsuya Yamamoto, Mitsuhiro Ito, Hiroshi Matsuoka, Hironobu Minami

**Affiliations:** ^1^ Division of Medical Oncology and Hematology Department of Medicine Kobe University Hospital Kobe Japan; ^2^ Division of Cardiovascular Medicine Department of Internal Medicine Kobe University Graduate School of Medicine Kobe Japan; ^3^ Jerome Lipper Multiple Myeloma Center Department of Medical Oncology Dana‐Farber Cancer Institute Harvard Medical School Boston Massachusetts USA; ^4^ BioResource Center Kobe University Hospital Kobe Japan; ^5^ Laboratory of Hematology Division of Medical Biophysics Kobe University Graduate School of Health Sciences Kobe Japan

**Keywords:** cardiac disease, global longitudinal strain, hematopoietic stem cell transplantation, posttransplant complication

## Abstract

Global longitudinal strain (GLS), a new cardiac parameter measured by the speckle‐tracking method, is reportedly more sensitive than ejection fraction (EF) in detecting slight cardiac dysfunction in heart failure patients. We validated the utility of GLS in allogeneic hematopoietic stem cell transplantation (HSCT) patients during a long‐term follow‐up. Medical records of patients who underwent allogeneic HSCT between 2013 and 2020 were reviewed retrospectively. We evaluated the last echocardiography performed before transplantation and those performed annually during the 5 years after transplantation. We also investigated newly diagnosed cardiac events, which developed after HSCT. Among 85 patients, 22 used cardioprotective drugs. The median follow‐up duration in surviving patients was 54.1 months (range, 2.9–92.6 months). GLS significantly decreased year by year, and patients taking cardioprotective agents tended to have a better GLS at 5 years than at 3 years, while EF did not change. Fifteen patients developed newly diagnosed cardiac events. Multivariate analysis revealed that low GLS and high serum ferritin levels at baseline were independently associated with the development of cardiac events. Therefore, we need a continuous follow‐up of cardiac function by GLS and prescription of cardioprotective drugs might be considered for HSCT patients with low GLS. Further research is warranted.

## 1 INTRODUCTION

Recent advances in allogeneic hematopoietic stem cell transplantation (HSCT) have improved the survival of patients with hematological malignancies, but late‐onset cardiovascular dysfunction is still a major problem [1]. The rate of congestive heart failure after HSCT is 2% [1, 2], resulting in high mortality. Allogeneic HSCT patients are heavily pretreated with chemotherapy, including anthracycline and conditioning regimens, and sometimes experience graft‐versus‐host disease that might damage cardiac function [3]. Although ejection fraction (EF) is the most widely used parameter of cardiac function, heart failure with preserved EF (HFpEF) also exists, and the prognosis is nearly the same for both; the mortality rates were reported to be 65% versus 68% for patients diagnosed of HFpEF and heart failure with reduced EF (HFrEF) at 5 years, respectively [4].

Recently, global longitudinal strain (GLS) has been reported to be more sensitive than EF in detecting cardiac dysfunction [5]. GLS is a simple, objective, and innovative assessment of cardiac function based on the speckle‐tracking method. Over the past few years, several studies on significance of GLS in heart failure patients or patients receiving chemotherapy have been reported [6, 7]. However, the validity of GLS in monitoring patients who undergo allogeneic HSCT remains to be evaluated.

The main aim of our study was to evaluate the long‐term cardiac function in patients who underwent allogeneic HSCT by GLS and EF using echocardiography.

## 2 METHODS

### 2.1 Patients

Medical records of all patients with various hematological disorders, who underwent allogeneic HSCT between April 2013 and March 2020 at Kobe University Hospital, were reviewed retrospectively. We evaluated the last echocardiography performed before transplantation, and those performed annually during the 5 years after transplantation. All 2D speckle‐tracking strain analyses were performed using GLS (AutoSTRAIN; TOMTEC ARENA, TOMTEC Imaging Systems GmbH, Munich, Germany). We selected three‐chamber patterns (two chambers, three chambers, and four chambers), and this software automatically traced the left ventricular endocardium and calculated the GLS. If the wrong region of interest was described to a deficit view, it was manually corrected; otherwise, one strain or the mean of two available strains was adopted.

We also investigated newly diagnosed cardiac events following HSCT. Cardiac events were defined as the development of myocardial infarction, arrhythmia, heart failure, which requires medication or intervention, and death caused by cardiovascular disease.

Furthermore, we analyzed the effect of cardioprotective agents such as beta‐blockers, angiotensin‐converting enzyme inhibitors (ACEI), and angiotensin receptor blockers (ARB) on cardiac function.

### 2.2 Statistical analysis

The EF and GLS parameters were compared using the Wilcoxon signed‐rank test. Overall survival (OS) curves were estimated using the Kaplan–Meier method and compared using the log‐rank test. The cumulative incidence of cardiac events was estimated using cumulative incidence functions, considering death as a competing risk, and Gray's test was performed. Univariate logistic analysis to identify the risk factors for cardiac events was followed by a multivariate logistic regression model using stepwise selection, with P levels for entry set at < 0.1. For all steps, statistical significance was set at *p* < 0.05. Moreover, relationship with each risk factor was calculated by Spearman rank correlation coefficient. All analyses were performed using EZR (Saitama Medical Center, Jichi Medical University, Saitama, Japan), a graphical user interface for R (The R Foundation for Statistical Computing, Vienna) [8].

## 3 RESULTS

### 3.1 Patient characteristics

In total, 85 patients who underwent allogeneic HSCT were included in this study, and their baseline characteristics are summarized in Table 1. Patients with relapsed or refractory acute myeloid leukemia received higher anthracycline doses, while patients with natural killer/T‐cell lymphoma, aplastic anemia, and myelodysplastic syndrome did not receive anthracycline.

**TABLE 1:** Patient characteristics

Median age (range, years)	49 (17–69)	
Median ferritin (range, ng/ml)	1147 (42–23163)	
**Sex**	***n***	**%**
Female	39	46
Male	46	54
**Disease**		
Acute myeloid leukemia	37	44
Acute lymphoblastic leukemia	13	15
Myelodysplastic syndrome	5	6
Malignant lymphoma	22	26
Others	8	9
**Stem cell source**		
Related donor (*n* = 24)		
Bone marrow	10	12
Peripheral blood	14	16
Unrelated donor (*n* = 61)		
Bone marrow	25	29
Peripheral blood	4	5
Cord blood	32	38
**Conditioning regimen**		
MAC, myeloablative conditioning	43	51
RIC, reduced‐intensity conditioning	42	49
**Total amount of anthracycline (mg/m^2^)**		
0	20	23
<200	22	26
≥200	43	51
**Ferritin (ng/dl)**		
No result	8	9
≤1500	45	53
>1500	32	38
**Reason for cardioprotective drugs use**	*n* = 22	
Hypertension	3	14
Decreased ejection fraction	12	55
Tachycardia	6	27
Others	1	4
**Timing of cardioprotective drugs started**	*n* = 22	
>3 months before transplantation	2	9
<3 months before transplantation	4	18
<1 year after transplantation	13	59
1–5 year(s) after transplantation	1	5
>5‐year after transplantation	2	9

The definition of “MAC” and “RIC” was based on the internationally recognized criteria in which “MAC” was defined as >8 Gy of fractionated total body irradiation (TBI), >5 Gy of single TBI, >6.4 mg/kg of intravenous busulfan, otherwise categorized as “RIC”.

Twenty‐two patients received cardioprotective drugs including beta‐blockers (*n* = 17) and ACEI or ARB (*n* = 8). With the exception of one patient who had idiopathic dilated cardiomyopathy before chemotherapy, all patients had been recently prescribed medication due to hypertension (*n* = 3), decreased EF (*n* = 12), or tachycardia, such as atrial fibrillation (*n* = 6). None had ischemic cardiac disease. Drug prescription or selection depends on the physician's choice. The timing for drug prescriptions was more than 3 months prior to HSCT (*n* = 2), within 3 months prior to HSCT (*n* = 4), within 1 year after HSCT (*n* = 13), within 5 years after HSCT (*n* = 1), and more than 5 years after HSCT (*n* = 2).

### 3.2 Evaluation of EF and GLS

**FIGURE 1:**
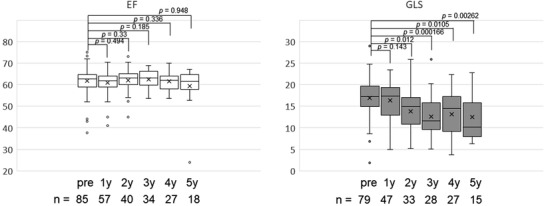
Ejection fraction (EF) and global longitudinal strain (GLS) over time. One year after transplantation, they were equivalent to those at baseline. However, 3 years after the transplantation, GLS was significantly lower than that at the baseline (pre).

**FIGURE 2:**
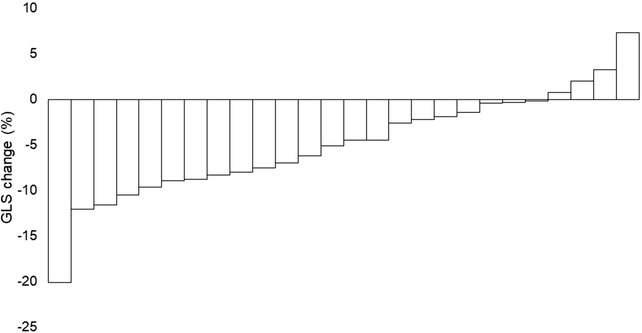
Waterfall chart of global longitudinal strain (GLS) change. GLS changes before and 3 years after the transplantation. GLS decreased in more than 80% of patients for whom data were available.

**FIGURE 3:**
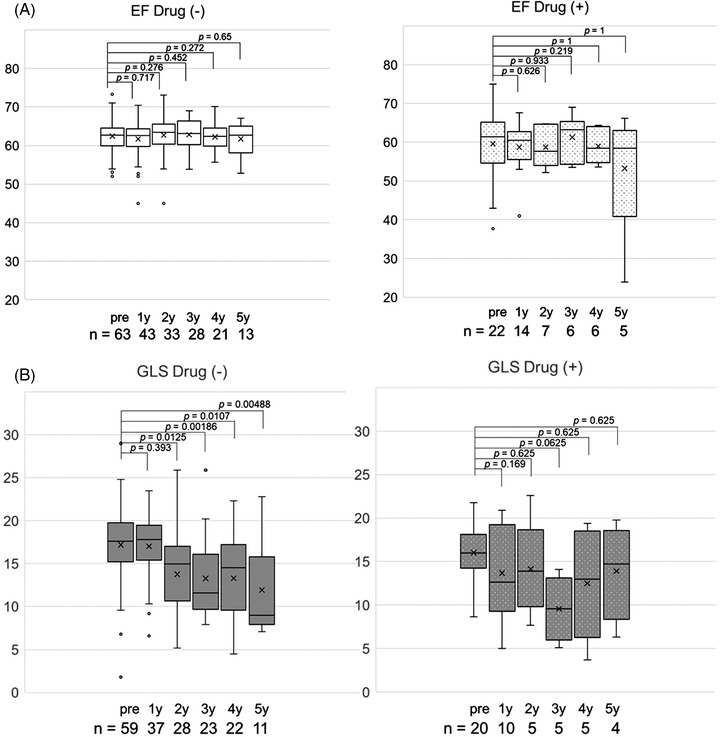
Ejection fraction (EF) and global longitudinal strain (GLS) grouped by patients with or without cardioprotective agents. (A) There were no EF changes in both groups. (B) Patients taking cardioprotective agents tended to have slightly better GLS at 5 years than that at 3 years. Among patients not taking cardioprotective agents, GLS gradually decreased over years.

The median EF and GLS at baseline were 62.6% (range, 37.7%–75%) and 17.6% (1.8%–29%), respectively. EF in each year after transplantation was equivalent to that at baseline. However, GLS significantly decreased compared to baseline, except for 1 year after transplantation (Figure 1). The median decrease in GLS between baseline and 3 years after transplantation was 4.8% (range; −7.3–20.1), and 15% of the patients (4 of 26 patients) experienced more than 10% decrease in GLS (Figure 2). Although the difference was not significant, patients taking cardioprotective agents (*n* = 22) tended to have slightly better GLS at 5 years than at 3 years; the median GLS changed from 9.6% at 3 years to 14.8% at 5 years (*p* = 0.25), while it decreased from 11.6% to 9.0% in the others who did not take cardioprotective agents (*p* = 0.20) (Figure 3). However, EF did not show any changes in both populations.

### 3.3 New‐onset cardiac events

**FIGURE 4:**
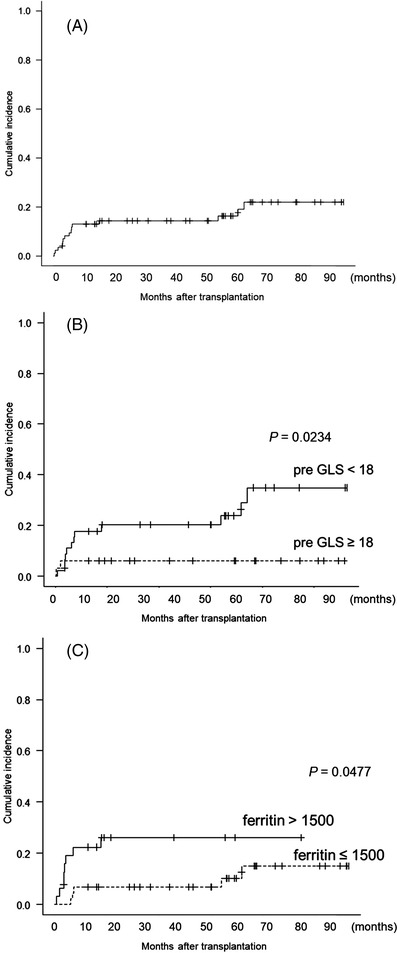
The cumulative incidence of newly diagnosed cardiac events that occurred both within 1 year and around 4 years after the transplant. (A) All patients, (B) GLS (≥18 vs. <18), and (C) serum ferritin level (>1500 ng/dl vs. ≤1500 ng/dl)

Of the 85 patients, 15 (18%) developed newly diagnosed cardiac events, which occurred bimodally (Figure 4A); 12 patients developed cardiac events approximately 1 year after transplantation; the others developed events beyond 4 years. Univariate analysis showed that patients with poor GLS at baseline (GLS < 18, *n* = 46) developed significantly more cardiac events (hazard ratio [HR] 4.75, 95% confidence interval [CI, 1.01–22.2], *p* = 0.048), and those with high levels of serum ferritin (>1500 ng/dl, *n* = 32), and had received more anthracycline (≥200 mg/m2, *n* = 43) tended to develop more cardiac events (HR 2.82, 95% CI [0.971–8.16], *p* = 0.057 and HR 3.00, 95% CI [0.948–9.49], *p* = 0.062, respectively; Table 2). The cumulative incidence curves of cardiac events grouped by GLS at baseline and serum ferritin levels are shown in Figure 4B,C, respectively. Multivariate analysis revealed that poor GLS at baseline and high levels of serum ferritin were independently associated with the development of cardiac events (HR 8.73, 95% CI [1.06–71.9], *p* = 0.044 and HR 3.70, 95% CI [1.22–11.2], *p* = 0.021, r = −0.09, *p* = 0.457, Table 2).

**TABLE 2:** Univariate/multivariate analysis for cardiac events

	Univariate			Multivariate		
	HR for cardiac events	95% CI	*p‐*Value	HR for cardiac events	95% CI	*p*‐Value
GLS at baseline < 18	4.75	1.01–22.2	0.048	8.73	1.06–71.9	0.044
Ferritin > 1500 ng/ml	2.82	0.971–8.16	0.057	3.70	1.22–11.2	0.021
Anthracycline ≥ 200 mg/m^2^	3.00	0.948–9.49	0.062			
QTc	0.99	0.980–1.01	0.51			
HCT‐CI	1.04	0.901–1.20	0.59			
Age	1.01	0.976–1.04	0.61			
Sex	1.24	0.447–3.46	0.68			
EF at baseline < 55	1.26	0.290–5.49	0.76			
Conditioning	0.89	0.329–2.43	0.83			

Abbreviations: CI, confidence interval; EF, ejection fraction; GLS, global longitudinal strain; HCT‐CI, hematopoietic stem cell transplantation‐comorbidity index; HR, hazard ratio.

### 3.4 Overall survival and mortality

The median follow‐up duration in surviving patients was 54.1 months (range, 2.9–92.6 months). The OS rate at 5 years was 54.6%, and almost all deaths occurred within 1 year after transplantation. The major causes of death were disease progression (*n* = 21), infection (*n* = 6), acute respiratory distress syndrome (*n* = 4), and others (*n* = 7). None of the patients were hospitalized or died of cardiac events. Although GLS at baseline was not associated with OS (OS at 5 years, 54% vs. 56%, *p* = 0.71), patients with a high serum ferritin level (>1500 ng/dl) had a significantly poor prognosis, as analyzed by the log‐rank test (OS at 5 years: 29% vs. 73%, *p* = 0.0017; Figures S1 and S2).

## 4 DISCUSSION

In this study, we found that GLS significantly decreased over time after transplantation, although the decrease in EF was not apparent. Additionally, less than 18% of GLS at baseline and serum ferritin levels of >1500 ng/dl were factors associated with more cardiac events after allogeneic HSCT. Several reports revealed a relationship between high ferritin and cardiac events in thalassemia patients [9, 10, 11]. Telfer et al. emphasized that most complications can be avoided if ferritin levels can be brought down to <1500 μg/L [10]. Iron is a catalyst generating oxidative stress, which can harm cardiac function because of a lot of oxidative consumption in cardiomyocytes. Besides, anthracycline may damage cardiac function by the formation of free radicals; iron overload may promote free radical damage [11]. Similarly, for OS, high levels of serum ferritin were significantly associated with poor outcomes [12], but the same was not true for GLS. Anthracycline is widely used to treat hematologic malignancies and is known to cause chemotherapy‐related cardiac dysfunction (CTRCD) [13, 14]. In our study, patients who had received ≥200 anthracycline had a tendency to develop more cardiac events (*p* = 0.06).

Several studies on GLS in allogeneic HSCT recipients have been reported [14, 15]; however, much remains to be elucidated regarding long‐term cardiac follow‐up with GLS. Recent studies have shown that GLS is a more sensitive parameter than EF for evaluating cardiac function in patients with heart failure or CTRCD [6, 7, 16]. The utility of GLS in allogeneic HSCT remains to be investigated. Our data indicated that GLS was more helpful than EF for long‐term cardiac follow‐up after allogeneic HSCT. Surprisingly, 46 of 85 (56%) patients had decreased GLS (<18) at baseline despite having a normal EF. It has been reported that GLS less than 18 is a high mortality factor in cancer patients [17], indicating HSCT patients are often at high risk for mortality. Our study highlights the need to evaluate cardiac function continuously using GLS.

The relationship between GLS reduction and cardiotoxicity has been reported for this decade; in particular, a 10%–15% early reduction in GLS appears to be the most useful parameter for the prediction of cardiotoxicity [18]. Our data included approximately 15% of patients who experienced a >10% reduction in GLS and might have some cardiac dysfunction in the future. We also found that cardioprotective drugs might improve GLS, although only four patients using them were followed for 5 years. Some researchers have recommended cardioprotective drugs for patients receiving chemotherapy because of their ability to improve cardiac function [16, 19]; however, the long‐term effectiveness of cardioprotective agents in HSCT patients is unknown. Our data may support the benefits of cardioprotective agents in HSCT patients. Prospective randomized studies are needed in the future to gather more evidence on this.

Our study has several limitations. First, it was retrospective in nature with relatively small sample size. Second, there were some cases in which the EF or GLS could not be calculated because of poor echocardiograms. However, this study is one of the few studies focused on the continuous utility of GLS in the long‐term cardiac follow‐up of HSCT patients. Additionally, the number of patients taking cardioprotective drugs before HSCT was too small to evaluate the potential effects for prevention of cardiac events.

EF has been used to assess cardiac function in patients undergoing allogeneic HSCT. Our study found that GLS after allogeneic HSCT significantly decreased, whereas EF did not change. Poor GLS and high serum ferritin levels before transplantation were significantly associated with the development of newly diagnosed cardiac events after several years. For long‐term follow‐up of HSCT patients, cardiac function should be monitored not by EF but by GLS, and prescribing cardioprotective drugs might be considered in HSCT patients with low GLS, further research is warranted.

## AUTHOR CONTRIBUTIONS

M.W. and K.Y. designed research, M.W. collected data, M.W. and K.Y. analyzed data, and M.W. wrote the paper. H.T. and H.M. supervised. The other all co‐authors reviewed the paper.

## CONFLICT OF INTEREST

Conflict of interest to declare is as follows; No one obtained financial support related to this study. Hironobu Minami received lecture fees from Daiichi Sankyo and Ono Pharmaceutical; Hidekazu Tanaka from AstraZeneca and Ono Pharmaceutical; Kimikazu Yakushijin from Nippon shinyaku and Pfizer. Hironobu Minami received research funding from Biopharma, Bayer, Incite, Ono, Daiichi Sankyo, Pfizer, Chugai, Bristol Myers Squibb, and Novartis; Kimikazu Yakushijin from Chugai, Jazz pharma, and PPD SNBL; Hiroshi Matsuoka from Novartis. Hironobu Minami received scholarship donation from Astelas, Bayer, Daiichi Sankyo, Bristol Myers Squibb, Chugai, Lilly, Pfizer, Ono Pharmaceutical, Taiho Pharma, Takeda, and Kyowa Kirin.

## FUNDING INFORMATION

The authors received no specific funding for this work.

## ETHICS STATEMENT

This retrospective study was approved by the Kobe University Hospital Ethics Committee (number: B210230), and the requirement for informed consent was waived. We provided the patients in this study the opportunity to opt out. This study was conducted in accordance with the Declaration of Helsinki.

## Supporting information

Supporting Information

## Data Availability

The data supporting the findings of this study are available from the corresponding author upon reasonable request.
