# Supplementary material for: Global longitudinal strain is superior to ejection fraction for long‐term follow‐up after allogeneic hematopoietic stem cell transplantation
Source: EJHaem. 2022 Nov 7;4(1):192–8. doi: 10.1002/jha2.586 (PMC9928647; doi:10.1002/jha2.586)

**Supplement figure 1: The overall survival grouped by GLS.**

The overall survival curves were grouped according to GLS (> 18 vs. < 18). There were no significant differences between the two groups.


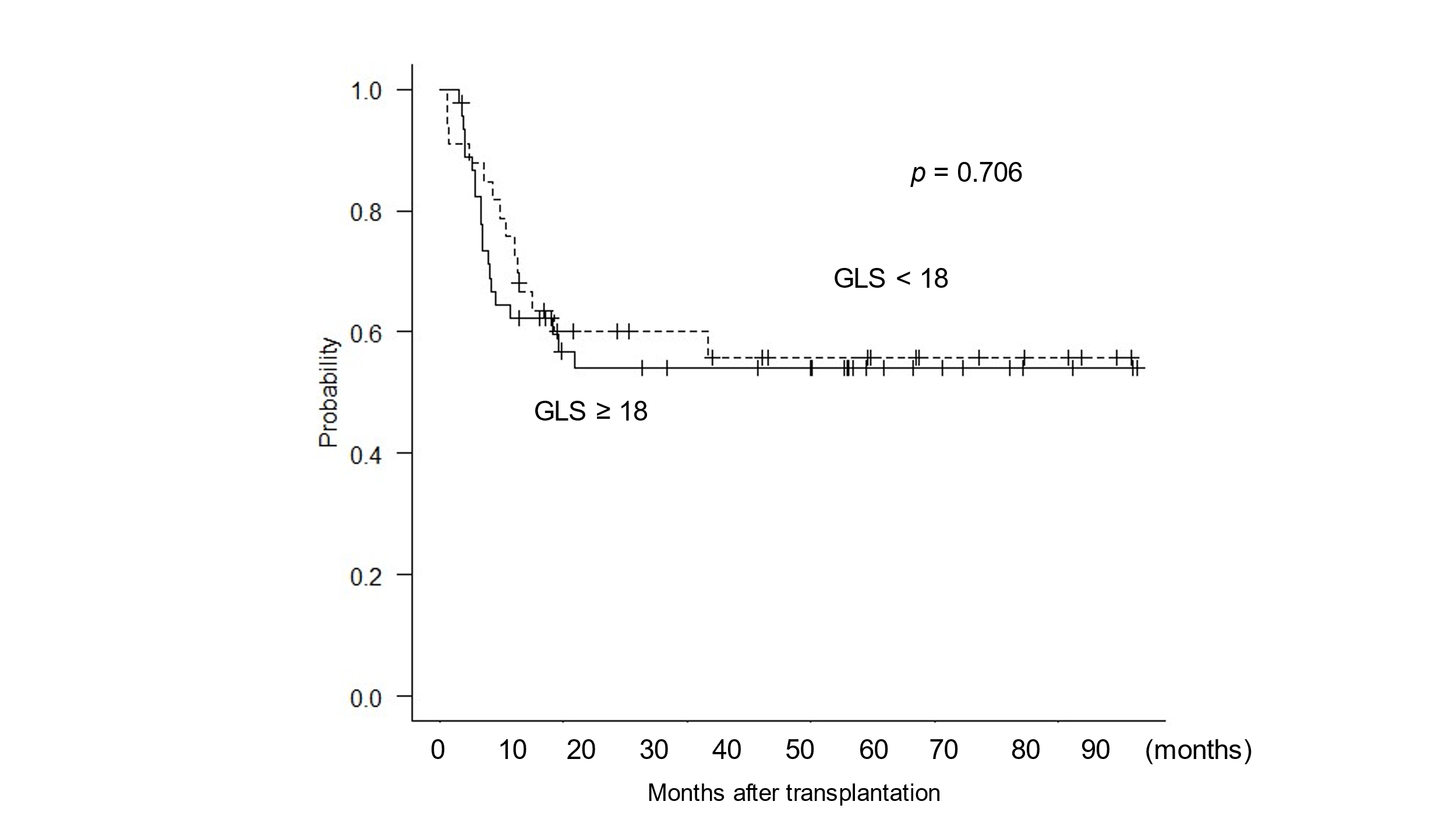


**Supplement figure 2: The overall survival grouped by serum ferritin levels.**

The overall survival curves were grouped according to serum ferritin levels (> 1500 ng/dL vs. < 1500 ng/dL).


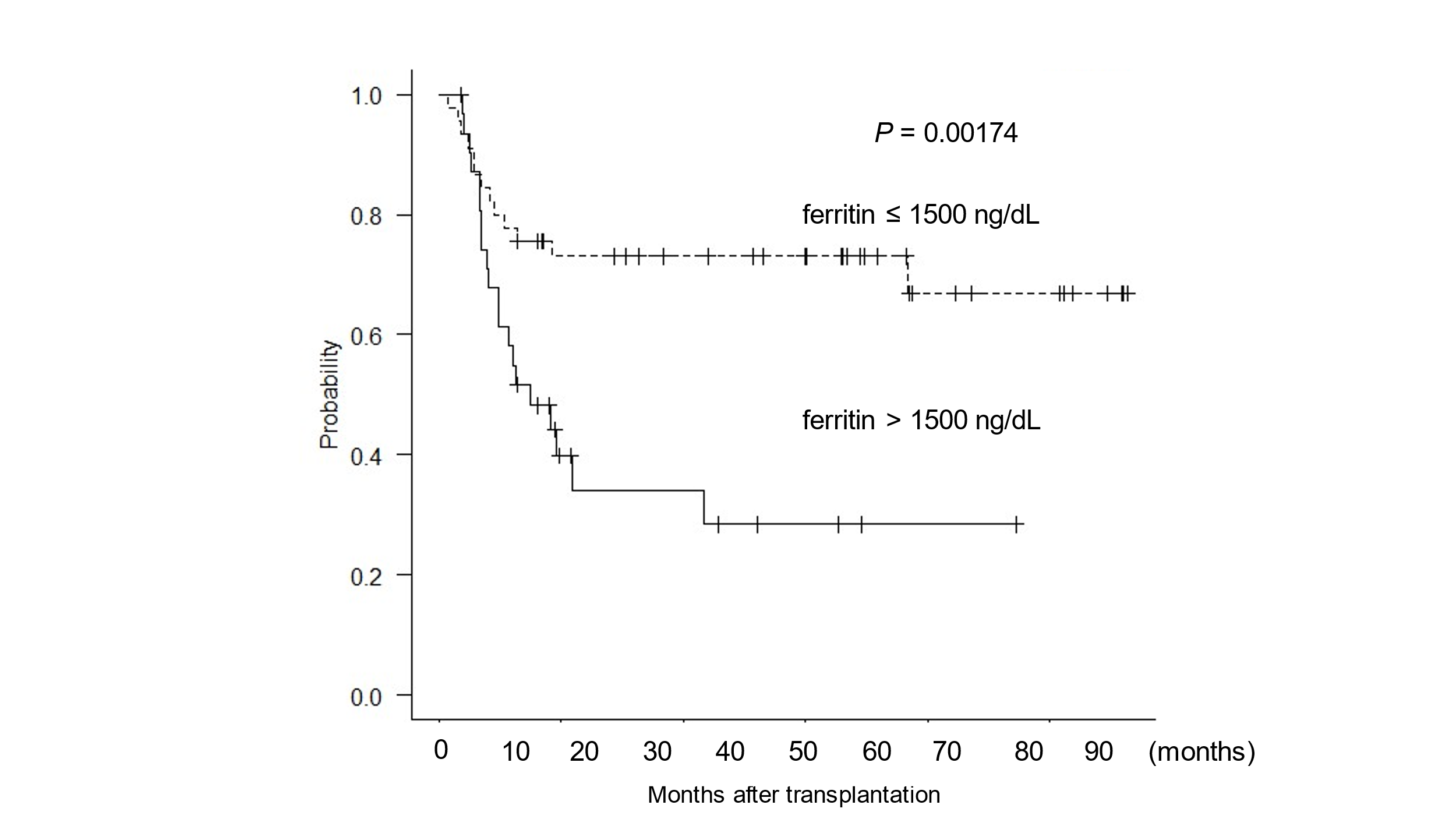

Supplement: Supplementary file 1 — Supporting Information [file JHA2-4-192-s001.docx]
